# Increased temperature, but not acidification, enhances fertilization and development in a tropical urchin: potential for adaptation to a tropicalized eastern Australia

**DOI:** 10.1111/eva.12218

**Published:** 2014-10-15

**Authors:** Shawna A Foo, Symon A Dworjanyn, Mehar S Khatkar, Alistair G B Poore, Maria Byrne

**Affiliations:** 1 School of Medical Sciences, The University of Sydney and Sydney Institute of Marine ScienceSydney, NSW, Australia; 2 National Marine Science Centre, Southern Cross UniversityCoffs Harbour, NSW, Australia; 3 Faculty of Veterinary Science, The University of SydneySydney, NSW, Australia; 4 Evolution and Ecology Research Centre, School of Biological, Earth and Environmental Sciences, University of New South WalesSydney, NSW, Australia; 5 Schools of Medical and Biological Sciences, The University of SydneySydney, NSW, Australia

**Keywords:** climate change, North Carolina II, ocean acidification, quantitative genetics, sea urchin

## Abstract

To predict the effects of global change on marine populations, it is important to measure the effects of climate stressors on performance and potential for adaptation. Adaptation depends on heritable genetic variance for stress tolerance being present in populations. We determined the effects of near-future ocean conditions on fertilization success of the sea urchin *Pseudoboletia indiana*. In 16 multiple dam-sire crosses, we quantified genetic variation in tolerance of warming (+3°C) and acidification (−0.3 to 0.5 pH units) at the gastrulation stage. Ocean acidification decreased fertilization across all dam-sire combinations with effects of pH significantly differing among the pairings. Decreased pH reduced the percentage of normal gastrulae with negative effects alleviated by increased temperature. Significant sire by environment interactions indicated the presence of heritable variation in tolerance of stressors at gastrulation and thus the potential for selection of resistant genotypes, which may enhance population persistence. A low genetic correlation indicated that genotypes that performed well at gastrulation in low pH did not necessarily perform well at higher temperatures. Furthermore, performance at fertilization was not necessarily a good predictor of performance at the later stage of gastrulation. Southern range edge populations of *Pseudoboletia indiana* may benefit from future warming with potential for extension of their distribution in south-east Australia.

## Introduction

Anthropogenic CO_2_ emissions are causing the climate in the ocean to change at an unprecedented rate (Doney et al. 2012). Many studies show the deleterious effects of concurrent ocean warming and acidification on early development of marine organisms (reviews: Byrne 2011; Byrne and Przeslawski 2013). Populations can respond to climate change through shifts in distribution, phenotypic plasticity and/or genetic adaptation or otherwise risk extinction. Predicting the prospects for long-term persistence in marine populations requires a better understanding of the capacity for phenotypic adjustment, a plastic response to environmental changes, and adaptation, a genetic response (Yeh and Price 2004; Gienapp et al. 2008; Hoffmann and Sgro 2011; Hansen et al. 2012).

Shifts in distribution allow species to track favourable environmental conditions. In response to ocean warming, poleward range shifts have been observed in marine species including fish, plankton, sea urchins and macroalgae (Perry et al. 2005; Parmesan 2006; Ling et al. 2009; Wernberg et al. 2011; Poloczanska et al. 2013). Some species, however, have a limited ability to shift their distributions in the time frames needed due to constraints such as limited dispersal potential, long generation times and lack of suitable habitat to migrate to (Hansen et al. 2012). For these species, the ability to produce multiple phenotypes under different conditions (phenotypic plasticity) can facilitate persistence in changing environments (Scheiner 1993; Via et al. 1995). Phenotypic plasticity may thus convey short-term tolerance to climate change stressors providing a temporal window for adaptive genetic change to occur (Thompson 1991; Chevin et al. 2010). Long-term persistence of populations will likely depend on genetic adaptation in the face of ocean change.

To predict whether marine populations will persist, it is important to determine the effects of ocean change stressors on performance and the potential for adaptation which is dependent on the levels of heritable variation for stress tolerance. Taxa such as sea urchins where male and female gametes can be isolated for experimental matings, provide a tractable and controllable model system for quantifying the contribution of heritable genetic variation to the overall phenotypic variation. Sires are considered to only contribute genetic effects to offspring performance (but see Crean et al. 2013), and so sire x environment interactions can be used to determine the presence of additive genetic variation in the response of offspring to environmental change (Lynch and Walsh 1998). On the other hand, dam effects include both additive genetic effects and environmental effects such as variation in egg provisioning (Mousseau and Fox 1998).

Despite the clear need to understand genetic variation in stress tolerance present in marine populations (Munday et al. 2013; Sunday et al. 2014), relatively few studies have used the tools of quantitative genetics in global change studies with marine organisms. Results to date indicate mixed outcomes for populations with respect to the presence of additive genetic variation in response to stressful environments. The copepod *Tigriopus californicus* showed little adaptive potential in response to a selection regime of increased temperature (Kelly et al. 2012). Similarly, the bryozoan, *Celleporaria nodulosa* showed no variation in tolerance to temperature and pH among clones (Durrant et al. 2013). On the other hand, studies on sea urchins, mussels, bryozoans and macroalgae have found significant levels of variation among genotypes, providing the potential for adaptation to ocean warming and acidification (Pistevos et al. 2011; Sunday et al. 2011; Foo et al. 2012; Kelly et al. 2013; Clark et al. 2013). These studies have largely investigated adaptation to a single stressor (temperature: Meyer et al. 2009; Kelly et al. 2012; Clark et al. 2013; acidification: Sunday et al. 2011; Kelly et al. 2013; Pespeni et al. 2013; Sunday et al. 2014) with three studies investigating the response to both stressors concurrently (Pistevos et al. 2011; Foo et al. 2012; Durrant et al. 2013). Thus, we have limited understanding of possible interactions between genotypes and multiple stressors.

In this study, the potential for adaptation to increased temperature and acidification in the tropical sea urchin *Pseudoboletia indiana* was investigated with a quantitative genetics approach using male-female crosses in all combinations of parents under warming-acidification regimes. This species has a broad Indo-Pacific distribution, from Madagascar to Hawaii and Easter Island and from Japan to Australia (Turner and Graham 2003). With its recent poleward extension into the Tasman Sea (Pope 1964; Australian Museum records), *P. indiana* is also found in the warm temperate waters of Sydney Harbour representing its southern range end. In east Australia, poleward range extension of tropical species is occurring due to increased southerly flow of the East Australian Current, and as temperature continues to rise, *P. indiana* and other tropical species have the potential to migrate poleward in this region and elsewhere (Johnson et al. 2011; Sunday et al. 2014). The impact of increased temperature on echinoderm development is well understood, especially for temperate species (Review: Byrne 2010). For several Australian temperate sea urchin species, a 3°C increase in temperature is deleterious to early development (Byrne 2012; Foo et al. 2012). Therefore, it is of interest to assess the effects of regional ocean warming on populations of a tropical sea urchin species at its warm temperate edge.

The responses of echinoderm fertilization to increased acidification and temperature have been mixed with outcomes depending on the species and whether the experimental design incorporated multiple male and female parents (spawner population approach) or individual pair responses (Byrne 2011, 2012; Schlegal and Havenhand 2012). While experimental designs that pool multiple males and females find that fertilization is fairly robust to decreased pH, results with single male–female crosses are more variable (Foo et al. 2012; Schlegal and Havenhand 2012; Sewell et al. 2014). In sea urchins, fertilization is mediated by the protein bindin which controls sperm attachment to the egg (Vacquier and Moy 1977). Eggs show strong discrimination depending on male bindin genotype mating most successfully with sperm having a similar bindin genotype to the egg (Palumbi 1999; Zigler et al. 2008; Evans and Sherman 2013). Intense sperm competition at fertilization and differences in compatibility between parental haplotypes are nonadditive genetic differences and can explain the differences in experimental outcomes using multiple males/females versus individual pairs.

Here, we use the North Carolina II quantitative genetic design (Lynch and Walsh 1998), where sires and dams are mated in all combinations to determine if additive genetic variance underlies the tolerance of *P. indiana* embryos to ocean change scenarios predicted for this region for 2060 and beyond (Hobday and Lough 2011; IPCC 2013). Maternal influence wanes as the zygotic genome takes over in sea urchin development soon after fertilization (Hamdoun and Epel 2007; Tadros and Lipshitz 2009). Thus while fertilization was undertaken in experimental conditions to better reflect future scenarios, genetic performance was assessed at the embryo stage with respect to the contributions of sire and dam to determine if *P. indiana* has the additive genetic variance required to adapt changing ocean conditions. Furthermore, investigating the performance of genotypes across multiple environments, as in this study, allows calculation of genetic correlations among traits, the proportion of variance that two genetic traits share (Sgro and Blows 2004). We addressed the following questions (i) Is fertilization robust across future ocean change scenarios? (ii) Does the performance of pairs differ across the different treatments? (iii) Will the percentage of normal gastrulae be reduced in ocean warming and ocean acidification scenarios? (iv) How does performance of genotypes among treatments compare across two stages (fertilization and gastrulation) and (v) Will significant additive genetic variation (significant interactions of sire with warming and acidification treatments) facilitate persistence of *P. indiana*?

## Materials and methods

### Study species and collection sites

*Pseudoboletia indiana* was collected from 4 to 6 m depth at Camp Cove, Sydney Harbour, New South Wales (33°50′ 21.32S, 151°16′42.2E) in April 2013 during their peak spawning period (Zigler et al. 2008). Animals were transported in ambient seawater in a cool box and transferred to large flow through aquaria (80 L; 22°C) shortly after collection. They were used for experiments within days of collection. The temperature during the collection period, as indicated by sea surface temperature (SST) recordings during the spawning season, ranged between 21.5 to 22.5°C (http://www.metoc.gov.au/products/data/aussst.php). The animals were collected under permit (NSW DPI: P00/0015-6.0).

### Fertilization and the North Carolina II design

Spawning of *P. indiana* was induced by injection of 2–4 mL of 0.5 M KCl. Following routine procedure, eggs from each female were placed in separate beakers of fresh, filtered seawater (FSW; 1 *μ*m). Sperm from each male was stored dry at 4°C until use. Egg density was determined in counts of 100 mL aliquots from the egg suspension. Approximately 200 eggs were placed in rearing containers (100-mL glass jars) containing experimental seawater 20 min prior to fertilization. Thus, eggs were fertilized in experimental temperature/pH conditions (see below). Hemocytometer counts of semen samples diluted with experimental FSW were used to determine the amount of sperm solution required to achieve a final sperm to egg ratio of 500:1; 1 × 10^3^ sperm/mL. Eggs were fertilized with the sperm solutions and after 10 min, the water in each jar was changed to remove excess sperm and prevent polyspermy.

Single sire-dam crosses were carried out in two experimental runs (blocks) with each block using gametes from 2 dams and 4 sires crossed in all combinations. Each block thus resulted in 8 full-sib families (total of 16 families) and was run concurrently. Each family was exposed to each of the six combinations of pH and temperature treatments with three replicates for each family by treatment combination. Thus, each block had a total of 144 jars (2 females × 4 males × 3 pH levels × 2 temperature × 3 replicates). At 1 and 24 h, a haphazardly selected sample of approximately 50 embryos was pipetted from the containers, placed into tubes and fixed with 2% glutaraldehyde in FSW. The first 30–50 embryos haphazardly selected from each tube were examined microscopically (Leica, North Ryde, Australia) and scored for successful development. At 1 h, the percentage of successfully fertilized embryos was determined based on the presence of a fertilization envelope and/or cleavage. At 24 h, the percentage of gastrulae was calculated from counts of normal/abnormal and arrested embryos (see Gilbert 2000). The number of embryos arrested at fertilization (e.g. fertilization envelope only) was low (<1%), indicating that polyspermy was minimal.

### Experimental conditions

Experimental treatments consisted of two temperatures (Mean ± SE, control 22.08 ± 0.06°C and 25.04 ±0.04°C) and three pH_NIST_ levels (Mean ± SE, control 8.12 ± 0.004, 7.85 ± 0.031, and 7.69 ± 0.006) in all combinations (Table 1). Treatments were based on model projections for near-future (2060) surface ocean waters in the south-east Australia global change hot spot where SST have been warming appreciably for decades (Hobday and Lough 2011; IPCC 2013).

**Table 1:** Experimental conditions in experiments with *Pseudoboletia indiana*

	22°C			25°C		
	pH 8.1	pH 7.8	pH 7.6	pH 8.1	pH 7.8	pH 7.6
Temp	21.81 (0.02)	21.91 (0.01)	22.5 (0.02)	24.77 (0.05)	25.20 (0.00)	25.14 (0.02)
pH_T_	8.00 (0.02)	7.86 (0.02)	7.56 (0.03)	7.95 (0.04)	7.69 (0.02)	7.64 (0.01)
pH_NIST_	8.08 (0.00)	7.88 (0.00)	7.71 (0.01)	8.11 (0.00)	7.82 (0.00)	7.66 (0.01)
*p*CO_2_	347.75 (2.51)	616.93 (5.59)	923.72 (21.49)	319.00 (3.65)	706.15 (6.18)	1070.67 (14.86)
ΩCa	4.84 (0.02)	3.44 (0.02)	2.42 (0.05)	5.76 (0.04)	3.35 (0.02)	2.46 (0.03)
ΩAr	3.16 (0.02)	2.25 (0.02)	1.58 (0.03)	3.79 (0.02)	2.21 (0.01)	1.62 (0.02)

Mean values (±SE, *n* = 9) for pH_NIST_ measured daily per treatment is presented with pH_T_ (determined in CO2SYS using data for dissolved inorganic carbon (DIC) and TA) for comparison. pH_T_, *p*CO_2_ and the saturation states of calcite (Ω_ca_) and aragonite (Ω_ar_) were calculated in CO2SYS using data on DIC and total alkalinity (TA = 2258.1 ± 15.6 *μ*mol/kg, *n* = 12), salinity (34.1 ± 0.04, *n* = 12) and temperature for each treatment.

Filtered (1 *μ*m) experimental FSW was supplied from a flow through system (ambient pH_NIST_ 8.12, 22.1°C) at the Sydney Institute of Marine Science. Water temperature was controlled by thermal mixers supplying 80 L header tanks. Experimental pH was controlled via a mixed CO_2_ supply where a pH controller (Parker, Castle Hill, Australia) regulated the amount of CO_2_ gas supplied into the airline. The required amount of air and CO_2_ was bubbled through ceramic diffusers into the 80 L header tanks controlled by an automatic CO_2_ injection and *p*CO_2_ feedback system (BioSys custom system; http://www.biosysconsulting.com.au/index.html), set at ppm equivalent to pH 7.6 and pH 7.8. The controls were FSW at ambient temperature and pH.

Temperature, pH and salinity were measured in all treatments (*n* = 9 per treatment across both blocks) using a pH meter (WTW—Wissenschaftilich-TechnischeWerkstätten GmbG P4) and probe (WTW SenTix® 41 pH electrode; precision ± 0.01 pH units). These parameters were measured at the beginning of the experiment with the water used to fill jars and measured in 8 randomly selected jars from each treatment at the end of the experiment (24 h). The salinity of treatment water was 34 psu, and dissolved oxygen remained >90%. Probes were calibrated using NIST high precision buffers pH 4.0, 7.0 and 10.0 (ProSciTech, Thuringowa Central, Australia).

Water samples (250 mL) were collected at the beginning and conclusion of the experiment, filtered through a 0.45-mm syringe filter and fixed with 100 *μ*L of saturated HgCl. These were used to determine total alkalinity (TA) by potentiometric titration (Metrohm 888 Titrando) using certified reference standards (Dickson et al. 2007) and total dissolved carbon (TCO_2_) using the Apollo SciTech DIC Analyzer AS-C3 (http://www.apolloscitech.com/DIC.htm). Experimental *p*CO_2_ and pH_T_ (Table 1) were determined from TA, TCO_2_, temperature, pH_NIST_ and salinity data using CO2SYS (Pierrot et al. 2006) using the dissociation constants of Mehrbach et al. 1973 as refitted by Dickson and Millero 1987.

### Statistical analyses

Percentage fertilization and percentage of normal gastrulae data were analysed using analysis of variance (anova) with temperature and pH as fixed factors, experimental block as a random factor, and sire and dam as random factors nested within blocks. As some significance tests involved quasi F ratios [in which significance tests derived from the F distribution are unreliable (Quinn and Keough 2002)], we calculated significance of the F statistics using 9999 permutations of the raw data for all factors in the permanova routine of Primer V6 (Anderson et al. 2008). The assumptions of normality and homogeneity of variance were checked by frequency histograms of the residuals and scatter plots of residuals versus estimates. The distribution of the residuals was normal, and no transformation was necessary.

For the gastrulation trait, the stage by which the zygotic genome is fully operational (Tadros and Lipshitz 2009), reaction norms were plotted to visualize the interactions between male genotypes across a range of environments (Lynch and Walsh 1998). The genetic correlation of embryo performance (% of normal gastrulae) across temperature and pH environments was used to quantify the genotype x environment interaction using variance components derived from restricted error maximum likelihood (REML) estimates calculated in the R package lme4 (available at http://cran.rproject.org/web/packages/lme4/index.html). Variance components for the random factors were calculated in a single analysis with all factors [temperature, pH, block, males, females). Genetic correlations were calculated using the causal variance components associated with the sire effects (additive genetic (V_A_)] and the interaction effects between sires and each of the environmental factors of temperature (V_AT_), pH (V_A__pH_) and both temperature and pH (V_AT pH_). Genetic correlations for the same trait averaged over both types of environments (r*_G_), the genetic correlation for the same trait within one environmental class (i.e. temperature; r*_G(T)_) and the genetic correlation within the other environmental class (i.e. pH; r*_G(pH)_) were calculated using equations from Eisen and Saxton (1983):


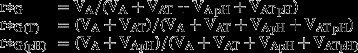


Linear regression analyses were performed to assess the relationship between performance across the two different life history stages; fertilization and gastrulation using percentage performance data for each pair over both stages over the six treatments.

Heritability was estimated using animal, sire and dam models for fertilization and gastrulation data across all treatments (Knott et al. 1995; Lynch and Walsh 1998). The animal model considers all relationships in the pedigree and computes additive genetic variance based on the additive genetic relationship matrix. A detailed description and application of the animal model are given in Kruuk (2004). Multiple observations on the same genotype were included in the models as random effects and were used to compute repeatability (variation between replicates). Temperature and pH were fixed effects and block a random effect. The models were fitting using ASReml (Gilmour et al. 2009). Heritability estimates were also calculated for each treatment combination.

## Results

### Effects of increased temperature and decreased pH on fertilization and the importance of pair compatibility

**Figure 1:**
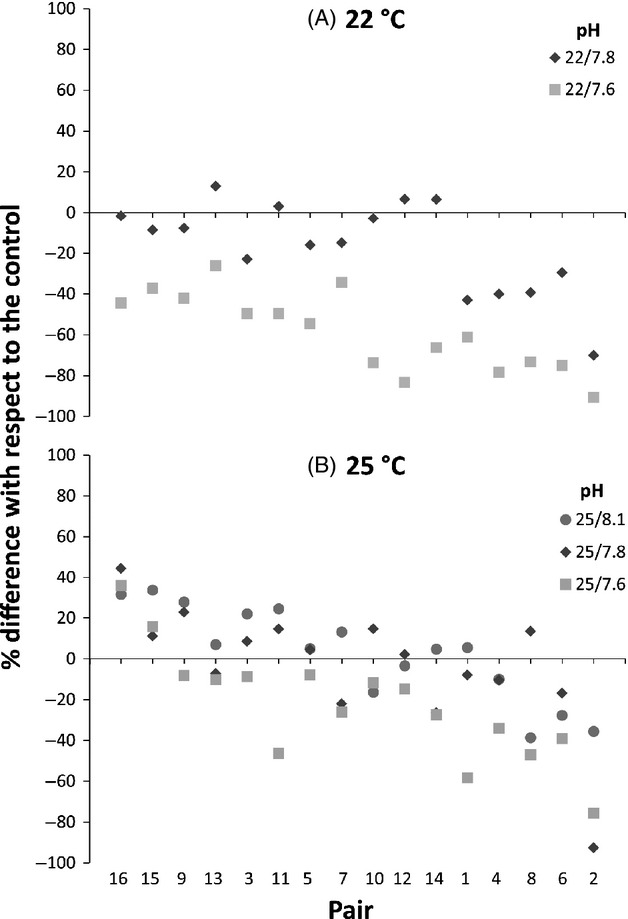
The difference in fertilization success with respect to the control treatment (22°C, pH 8.1) in 16 different male–female pairs across five experimental treatments. Mean fertilization success per genotype is displayed for the different pH levels across the control temperature (A) and increased temperature (B). Symbols above the line display higher fertilization success than the control, while fertilization success was lower than the control for those symbols below the line. Pairs are ranked from the best to the worst performing from left to right.

Fertilization success in the 16 sire/dam crosses for *Pseudoboletia indiana* ranged between 29% and 93% in the control conditions (mean of 63.5% ± SE 4.6). Decreased pH had a significant effect in reducing fertilization success, with temperature significantly increasing fertilization. Both factors were strongly influenced by male/female pairings as indicated by significant sire × dam × temp and sire × dam × pH interactions (Table 2; Fig. 1). The sire × dam × temp × pH interaction indicates the effect of increased temperature in reducing the negative effect of decreased pH on percentage fertilization (Table 2; Fig. 1). This is also evident in the scatter plot as seen in the comparison of fertilization in the 22°C/pH 7.6 and 25°C/7.6 treatments (Fig. 1). The most extreme pH treatment lowered fertilization success across all but two pairs showing the influence of gamete compatibility, resulting in different responses to the same treatment (Fig. 1).

**Table 2:** anova of percentage fertilisation data of *Pseudoboletia indiana*

Source	df	MS	*F*	*P*
Bl	1	94.712	3.35E-02	0.9998
Te	1	21958	13.714	0.1692
pH	2	45612	20.5	**0.047**
Ma(Bl)	6	1986.6	7.0254	**0.0193**
Fe(Bl)	2	9266.1	32.769	**0.0012**
Bl × Te	1	1601.1	0.76159	0.5789
Bl × pH	2	2225	2.3551	0.0837
Te × pH	2	6921.6	3.7581	0.2195
Ma(Bl) × Fe(Bl)	6	282.77	1.607	0.1482
Ma(Bl) × Te	6	1934.1	4.63	**0.0438**
Ma(Bl) × pH	12	893.52	1.6226	0.2046
Fe(Bl) × Te	2	716.66	1.7156	0.2522
Fe(Bl) × pH	4	285.05	0.51764	0.7227
Bl × Te × pH	2	1841.8	0.99945	0.4698
Ma(Bl) × Fe(Bl) × Te	6	417.73	2.374	**0.0341**
Ma(Bl) × Fe(Bl) × pH	12	550.68	3.1295	**0.0004**
Ma(Bl) × Te × pH	12	261.83	0.52167	0.8594
Fe(Bl) × Te × pH	4	2083.1	4.1505	**0.0257**
Ma(Bl) × Fe(Bl) × Te × pH	12	501.9	2.8523	**0.0013**
Res	192	175.96		

anova of fertilisation data of single dam-sire crosses across various temperature (Te) and pH conditions. These were fixed factors, with experimental block (Bl) as a random factor, and male (Ma) and female (Fe) identity as random factors nested within block. Significant effects are shown in bold (*P* < 0.05).

Sire and dam identity also influenced fertilization success with significant interactions between sire and temperature and between dam, temperature and pH (Table 2). The sire x temperature interaction indicates that the effect of +3°C varied among paternal half-siblings. Similarly, the effects of pH and temperature varied among maternal half-siblings with an increase in fertilization success at +3°C and a decrease with lowered pH (Table 2).

### Effects of increased temperature and decreased pH on gastrulation

**Figure 2:**
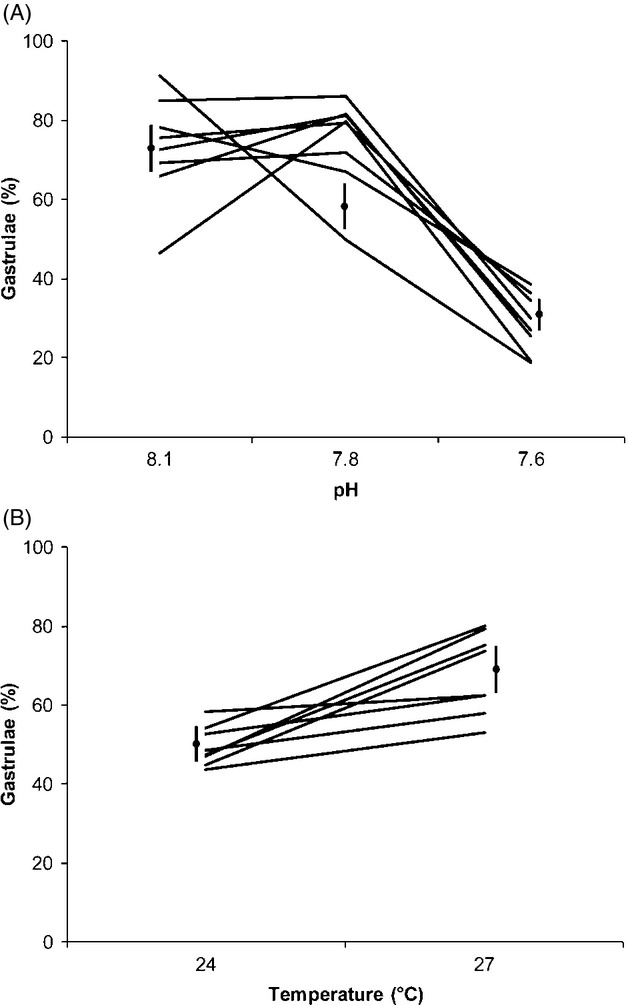
Reaction norms showing the responses of the progeny of eight male genotypes to increased temperature and reduced pH. The reaction norms show the percentage of normal gastrulae in experimental temperatures pooled for pH (A) and in experimental pH levels pooled for temperature (B). Lines represent the mean percentage of paternal half-siblings with standard errors indicated (*n* = 8).

Increased temperature significantly increased the percentage of normal gastrulae, with temperature increasing performance at the two decreased pH treatments but not at the control pH (Table 3). The effects of sire and dam on the percentage of normal gastrulae were significant as were the interactions between sire and temperature, and dam and temperature. The sire by environment interactions is illustrated in reaction norms showing the response of paternal half-siblings to pH and temperature treatments (Fig. 2). The significant sire x temperature interaction indicates that the effect of the +3°C treatment varied among paternal half-sib families, as shown by the different slopes in the reaction norms. Male and female compatibility was also important with significant sire × dam, sire × dam ×temp, sire × dam × pH, and sire × dam × temp × pH interactions (Table 3).

**Table 3:** anova of percentage of normal gastrulae of *Pseudoboletia indiana*

Source	df	MS	*F*	*P*
Bl	1	590.18	0.31628	0.8814
Te	1	23030	249.9	**0.0377**
pH	2	65919	15.575	0.058
Ma(Bl)	6	1422.8	8.056	0.0116
Fe(Bl)	2	1001.7	5.6715	**0.043**
Bl × Te	1	92.157	6.59E-02	0.9977
Bl × pH	2	4232.5	2.8429	**0.0464**
Te × pH	2	27451	919.88	**0.0019**
Ma(Bl) × Fe(Bl)	6	176.61	2.2257	**0.041**
Ma(Bl) × Te	6	1178.3	6.251	**0.0216**
Ma(Bl) × pH	12	1248	2.4223	0.0677
Fe(Bl) × Te	2	3079.1	16.336	**0.0037**
Fe(Bl) × pH	4	422	0.81907	0.53
Bl × Te × pH	2	29.841	0.65838	0.7135
Ma(Bl) × Fe(Bl) × Te	6	188.49	2.3754	**0.0296**
Ma(Bl) × Fe(Bl) × pH	12	515.22	6.493	**0.0001**
Ma(Bl) × Te × pH	12	280.2	0.39025	0.9441
Fe(Bl) × Te × pH	4	855.69	1.1918	0.3676
Ma(Bl) × Fe(Bl) × Te × pH	12	718	9.0486	**0.0001**
Res	192	79.35		

anova of gastrulation data of single dam-sire crosses across temperature (Te) and pH treatments. Temperature and pH are fixed factors, experimental block (Bl) a random factor, and male (Ma) and female (Fe) identity random factors nested within block. Significant effects are shown in bold (*P* < 0.05).

There was a low genetic correlation (r*_G_) of 0.1 in the gastrulation trait across all environments indicating that genotypes that performed well in a particular combination of temperatures and pH might not necessarily perform similarly in other environmental combinations. However, there were stronger positive genetic correlations across the three temperature levels (r*_G(t)_ = 0.47) and across the three pH levels (r*_G(pH)_ = 0.63). Thus, genotypes that performed well at control temperatures also performed the best in high temperatures and similarly for pH.

### Does performance at fertilization predict gastrulation success?

**Figure 3:**
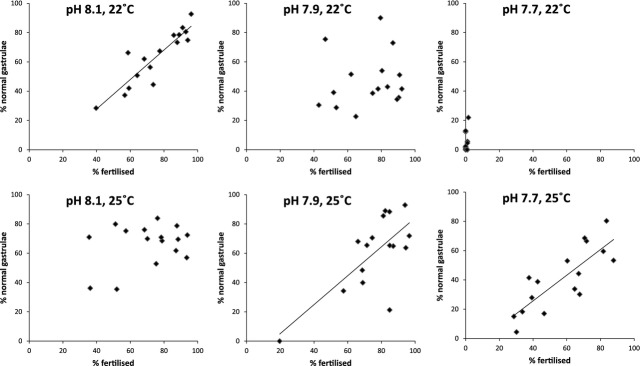
Scatter plots of the relationship between pair performance at fertilization (*y*-axis) and at gastrulation (*x*-axis). Each point represents the mean performance of an individual pair in each treatment across both stages. A positive relationship was evident for the control pH/control temp (*R*^2^ = 0.81, *P* = 0.000002), pH 7.8/+3°C (R^2^ = 0.51, *P* = 0.002) and pH 7.6/+3°C treatments (R^2^ = 0.66, *P* = 0.0001).

The relationships between fertilization success and percentage of normal gastrulae show that the pairs did not perform consistently across all environments (Fig. 3). A positive relationship was evident for the control pH/22.08°C, pH 7.9/+2.99°C and pH 7.7/+2.99°C environments. Here, genotypes that had a high percentage of fertilization also had the highest percentage of normal gastrulae. However, this does not hold true when decreased pH is considered in isolation. Thus, genotypes that perform well at fertilization were good genotypes at gastrulation, but only under certain pH/temperature conditions.

### Repeatability and heritability estimates for fertilization and gastrulation

The heritability estimate from the animal model at fertilization was moderate (0.222 ± 0.1), contributed mainly by the dam component as the heritability estimate from the sire component was zero (Table 4). Estimates of repeatability (0.0356 ± 0.0323) and heritability (0.062 ± 0.047) from the animal model for gastrulation were quite low, with the heritability based on sire component lower when compared to fertilization (0.094 ± 0.075) albeit with equally large standard error. Heritability estimates were also calculated for each of the six treatments. However, as estimates were quite variable across different models and between different treatments with comparatively large standard errors, these results are presented in the Supporting Information only (Table S1).

**Table 4:** Heritability estimates at fertilisation and gastrulation for *Pseudoboletia indiana*

Trait	Model	Parameter	Estimate	SE
Fertilisation	IntraFamily	Repeatability	0.1932	0.0721
Fertilisation	Animal	Heritability	0.2217	0.0951
Fertilisation	Sire	Heritability	0.1582	0.1079
Fertilisation	Dam	Heritability	0.3251	0.2461
Gastrula	IntraFamily	Repeatability	0.0356	0.0323
Gastrula	Animal	Heritability	0.0621	0.0472
Gastrula	Sire	Heritability	0.0904	0.0754
Gastrula	Dam	Heritability	0.0206	0.0423

Animal, sire and dam models were used to estimate heritability for fertilisation and gastrulation across all treatments. Multiple observations on the same genotype were included in the model as random effects and were used to compute repeatability. Temperature and pH were fixed effects and experimental block a random effect. The models were fitted using ASReml.

## Discussion

This study examined the performance of replicate male–female pairs of *Pseudoboletia indiana* at fertilization and the potential to adapt to climate change stressors at gastrulation. Decreased pH significantly reduced fertilization success, the magnitude of which depended on the individual pair, indicating the importance of prezygotic effects and the sperm bindin-egg bindin receptor system characteristic of sea urchin fertilization (Palumbi 1999). The percentage of normal gastrulae was reduced in ocean acidification scenarios but increased in warming scenarios. There was a slight positive genetic correlation between performance in increased temperature and decreased pH. Most importantly, the significant interaction between male and each stressor indicated the presence of additive genetic variance in the response of progeny to increased temperature and acidification. Interestingly, performance at fertilization did not necessarily predict performance at gastrulation.

### Fertilization success: prezygotic effects

For *P. indiana*, fertilization success decreased at low pH with significant influences of sire, dam and combination of sire and dam. For other sea urchin species using the multiple parent (population) approach (e.g. *Heliocidaris erythrogramma*, *H. tuberculata*), fertilization is fairly robust to future ocean change scenarios (Byrne 2012; Byrne and Przeslawski 2013). For *P. indiana*, this is not the case for single male-female crosses as also found for other sea urchin species using a similar approach (Schlegal and Havenhand 2012; Sewell et al. 2014) The multiple parent spawning approach does not allow detection of intraspecific variation. To allow a full assessment of offspring response to treatments, the genotype of the parents was taken into account, as in the design of our experiment. For *P. indiana*, none of the males consistently performed the best across all treatments, indicating there is no universally good male. Reduced pH can decrease the number of motile sperm (i.e. moving) in some sea urchins (Schlegal and Havenhand 2012) and this may have influenced the results with *P. indiana*. Swimming speed of sea urchin sperm is either not affected (Schlegal and Havenhand 2012) or actually enhanced (Caldwell et al. 2011) in ocean acidification scenarios. It should be noted that within-ejaculate variability of individual sperm, as shown to be important by Crean et al. (2012) where the offspring of the tunicate *Styela plicata* sired by longer-lived sperm performed better in control conditions, remains unexplored for *P. indiana*.

Although decreased pH had an overall negative effect on percentage fertilization, the scatter plot revealed that two pairs actually showed a positive response in decreased pH conditions with an increase of 36% and 16% compared to the control, also seen for *Sterechinus neumeyeri* and *Centrostephanus rodgersii* in response to acidification scenarios (Foo et al. 2012; Sewell et al. 2014). As nonadditive genetic differences (i.e. nonheritable) due to parental haplotype compatibility (e.g. sperm bindin-egg bindin receptor system) dominates the fertilization biology of sea urchins (Palumbi 1999; Zigler et al. 2008; Levitan and Ferrell 2006), this difference was expected.

Certain individuals may disproportionately contribute to the success of future generations (Schlegal and Havenhand 2012), although following through to gastrulation as in this study for *P. indiana* indicated that this is not straight forward. It is clearly important to follow through with zygotic (postfertilization) development to better understand adaptive potential (e.g. Sunday et al. 2011). Furthermore, this is supported by heritability estimates at fertilization where it can be seen that the dam contributes most to heritability at this stage compared to the sire component.

### Gastrulation: additive genetic variance, genetic correlations and adaptive potential

At gastrulation (a postzygotic trait), normal development decreased at low pH for all genotypes of *P. indiana* with warming significantly increasing the percentage of normal gastrulae. The interaction between pH and temperature indicated that warming somewhat alleviated the effects of decreased pH. If this was a single stressor study, one may have concluded that normal development could not occur in a decreased pH environment, but here, we show that coupled with increased temperature, normal development can occur. For the biota of eastern Australia, temperature is the more important and contemporary stressor (Hobday and Lough 2011) with negative effects of present day and near-future warming reported for several echinoderm species (Nguyen et al. 2012). Thus it is interesting that decreased pH is the more important negative stressor for *P. indiana*.

The significant contribution of sire to progeny performance and the interaction between sire and temperature and with pH at gastrulation indicates the presence of additive genetic variation in tolerance to ocean warming and acidification conditions. It can be seen that the offspring of some males were more strongly affected by acidification than others but only at increased temperatures. Selection mediated by increased temperature and acidification would be expected to favour the more tolerant *P. indiana* genotypes allowing species persistence in future ocean change conditions. Most importantly, the significant sire × pH ×temperature interaction and nonsignificant sire × pH interaction indicate that adaptation to ocean acidification would not occur in this species in isolation from ocean warming.

For many echinoderms, maternal effects result in variable egg quality likely due to phenotypic effects associated with the maternal nutritive history, egg nutrients and maternal environmental history (Byrne et al. 2008; Byrne 2011). Normal development in sea urchins is influenced by maternal transcripts which may be influenced by maternal stress history (Hamdoun and Epel 2007). Thus, the significant interaction between dam and temperature where eggs of some females were more susceptible to stressors than others could be due to both genotypic and environmental/phenotypic effects.

Development in *P. indiana* was enhanced in the +3°C treatments. If spawning time in this species is influenced by temperature, as appears the case for many sea urchins (Byrne 1999), there is potential that adult *P. indiana* may be able to track favourable temperature conditions for offspring, to spawn at times to facilitate developmental success, a phenotypic adjustment of reproduction. Phenological change in biological events such as spawning is a major response to marine global change as seen for diatoms, copepods and fish larvae (Edwards and Richardson 2004). Developmental plasticity may provide short-term tolerance to climate change allowing the time for adaptive evolution to occur (Sultan 2007). Furthermore, epigenetic (nongenetic inheritance) can also affect progeny's response to environmental change (Bonduriansky et al. 2011). The environmental conditions the parent experiences can influence parts of its phenotype that can affect the development of its progeny (Visser 2008; Bonduriansky and Day 2009).

There was a genetic correlation of close to zero for the gastrulation trait among all temperature and pH treatments. This means that the progeny of parents that performed the best in the lower pH environment did not necessarily mean they performed the best in the warmer environment. This may indicate that there is little overlap in the gene sets that contribute to genetic variation in performance in response to these two stressors. Thus, evolution is not constrained in adapting to both stressors simultaneously. A previous study on a temperate sea urchin species found a high positive genetic correlation indicating similar gene sets influence performance in both ocean acidification and warming environments, which could enhance the speed at which natural selection can occur (Foo et al. 2012). For *P. indiana*, there were positive genetic correlations among the three levels of pH indicating that genotypes that performed the best in the control conditions also performed the best in the stressed pH environment and similarly for the different temperature environments.

The presence of standing genetic variation as indicated by significant sire × stressor interactions, and absence of a trade-off between tolerance to both pH and temperature, contributes to the potential of *P. indiana* to adapt to concurrent ocean acidification and warming and adds to the resilience of this important species in a changing ocean. When comparing heritability estimates across both stages, it can be seen that the dam contribution is much larger at fertilization, suggesting that the prezygotic stage is dominated by maternal effects. Sire effects remained similar throughout both developmental stages. However, estimates, especially at gastrulation, were quite low with proportionately large standard errors. Moreover, there were large differences in the estimates from animal, sire and dam models. As estimates of heritability should be lower as compared to their respective repeatability estimates, these results suggest that more data incorporating a greater number of genotypes are required to obtain more precise estimates of heritability and to better understand the genetic architecture of these traits (Lynch and Walsh 1998).

### Linking performance across different life history stages

When comparing the performance of pairs across fertilization and gastrulation, there was a positive relationship for the control environment, and the two treatments with combined pH and increased temperature. Pairs that had the highest fertilization success in these environments also had the highest percentage of normal gastrulae. However, when decreased pH is considered in isolation, this does not hold true for pairs with performance being unpredictable.

It is often assumed that certain genotypes that perform the best will continue to have superior performance across all developmental stages, for example larger larvae in various marine taxa typically show higher performance as juveniles and adults (Marshall and Keough 2008). Here, we show, however, that pairs that perform best in prezygotic stages did not necessarily predict their performance in postzygotic stages, or in stressed conditions. This indicates the lack of connection between pair performance at both stages and shows that looking only at prezygotic effects (e.g. fertilization) cannot be used to predict performance. Here, we only considered the gastrulation trait and the disconnection between fertilization success and gastrulation success for some pairs highlights the importance of considering all developmental stages.

### Implications for *Pseudoboletia indiana* and the tropicalization of eastern Australia

The gastrula stage of many echinoderms is sensitive to warming with an increased temperature of 4°C above ambient being deleterious to many species (Byrne et al. 2010; Byrne 2011). In this study, however, *P. indiana* exhibited a higher percentage of normal gastrulation at 3°C above ambient, with this level of warming being beneficial to early development. Temperature is likely to have been an important factor in establishing populations of this tropical species at its southern range edge in Sydney. Elsewhere in its range, *P. indiana* experiences temperatures 28°C and above (Clark and Rowe 1971). In Sydney, this species spawns in summer and autumn at temperatures ranging from 22 to 23°C (Zigler et al. 2012). Although the cool temperature tolerance of development is not known, it seems likely that the population in Sydney may be living where temperatures are just warm enough for successful development. This is similar for newly established *Centrostephanus rodgersii* populations in Tasmania where they live at their lower limit of developmental tolerance (12°C) (Ling et al. 2009; Hardy et al. 2014).

Ocean warming may facilitate the success of *P. indiana* in the temperate waters of Eastern Australia and may provide an opportunity for poleward range extension as seen with other tropical echinoids and asteroids (Pecorino et al. 2013; Hardy et al. 2014). Tropical echinoids tend to have a broader developmental thermal tolerance compared with temperate species facilitating poleward range extension to cooler climes (Sunday et al. 2011; Hardy et al. 2014). Southerly expansion of *P. indiana* is possible as there appears to be suitable habitat for this rocky reef species. As the latitudinal distribution of many marine species is related to the thermal tolerance of planktonic stages, understanding species' potential for poleward range extension with respect to the thermal tolerance of development is key to understanding how the seascape will change with further warming and acidification as not all species are affected equally (Sunday et al. 2012).

In conclusion, early development in *P. indiana* was sensitive to acidification at pH 7.6 while warming (+3°C) alleviated the negative effects of acidification at gastrulation. Male/female compatibility significantly affected performance; however, the pairs that performed the best in control conditions did not necessarily perform the best in stressed environments. Furthermore, it was clear that performance of pairs across different developmental stages was particularly unpredictable in ocean acidification conditions. Our analyses revealed the presence of significant additive genetic variation underlying success at gastrulation in response to ocean acidification and warming scenarios. Furthermore, due to *P. indiana*'s increased performance in warmer conditions, this species has potential to expand its population in Sydney at its range edge and beyond. The presence of tolerant genotypes indicates that Sydney Harbour populations of *P. indiana* are resilient to +3°C and combined with the lack of a negative correlation between tolerance to both decreased pH and warming will contribute to the potential of early development in *P. indiana* to adapt to a changing ocean. In eastern Australia, the Sydney region approximates the southern range edge of many tropical sea urchin species (e.g. *Diadema* spp.) (Miskelly 2002). For these species and *P. indiana*, it would be expected that performance would be enhanced as the ocean warms, with potential for population expansion locally and extension of their distribution poleward.

## Data archiving statement

Data for this study are available from the Dryad Digital Repository: http://doi.org/10.5061/dryad.gd71g

## Supporting Information

**Table S1.** Heritability estimates at fertilisation and gastrulation for *Pseudoboletia indiana* separated across the six experimental treatments.
