# Supplementary material for: Increased temperature, but not acidification, enhances fertilization and development in a tropical urchin: potential for adaptation to a tropicalized eastern Australia
Source: Evol Appl. 2014 Oct 15;7(10):1226–37. doi: 10.1111/eva.12218 (PMC4275094; doi:10.1111/eva.12218)
Supplement: Supplementary file 1 — Table S1. Heritability estimates at fertilisation and gastrulation for Pseudoboletia indiana separated across the six experimental treatments. [file eva0007-1226-sd1.docx]

**Supplementary Material**

**Table 1. Heritability estimates at fertilisation and gastrulation for *Pseudoboletia indiana* separated across the six experimental treatments**

| *Cleavage stage embryos* | | | | |
| --- | --- | --- | --- | --- |
| **Treatment** | **Model** | **Parameter** | **Estimate** | **SE** |
| **22/8.1** | IntraFamily | Repeatability | 0.3481 | 0.2137 |
|  | Animal | Heritability | 0.4581 | 0.3077 |
|  | Sire | Heritability | 0.2324 | 0.2277 |
|  | Dam | Heritability | 0.9236 | 0.8808 |
| **22/7.8** | IntraFamily | Repeatability | 0.454 | 0.2189 |
|  | Animal | Heritability | 0.6504 | 0.1356 |
|  | Sire | Heritability | 0.5087 | 0.3415 |
|  | Dam | Heritability | 0.83 | 0.4861 |
| **22/7.6** | IntraFamily | Repeatability | 0.3454 | 0.2402 |
|  | Animal | Heritability | 0.3493 | 0.2912 |
|  | Sire | Heritability | 0 | 0 |
|  | Dam | Heritability | 1.0973 | 1.0709 |
| **25/8.1** | IntraFamily | Repeatability | 0.2956 | 0.1653 |
|  | Animal | Heritability | 0.3082 | 0.202 |
|  | Sire | Heritability | 0.2286 | 0.2898 |
|  | Dam | Heritability | 0.3915 | 0.3678 |
| **25/7.8** | IntraFamily | Repeatability | 0.7922 | 0.0792 |
|  | Animal | Heritability | 0 | 0 |
|  | Sire | Heritability | 0.509 | 0.6304 |
|  | Dam | Heritability | 0 | 0 |
| **25/7.6** | IntraFamily | Repeatability | 0.6547 | 0.1176 |
|  | Animal | Heritability | 0.6328 | 0.1623 |
|  | Sire | Heritability | 0.5942 | 0.3773 |
|  | Dam | Heritability | 0.6789 | 0.4653 |
| ***Gastrulae*** | |  |  |  |
| **22/8.1** | IntraFamily | Repeatability | 0.3648 | 0.2432 |
|  | Animal | Heritability | 0.5092 | 0.3646 |
|  | Sire | Heritability | 0.3431 | 0.3043 |
|  | Dam | Heritability | 0.8666 | 0.873 |
| **22/7.8** | IntraFamily | Repeatability | 0.7349 | 0.0966 |
|  | Animal | Heritability | 0.2946 | 0.3511 |
|  | Sire | Heritability | 0.4095 | 0.5361 |
|  | Dam | Heritability | 0.2108 | 0.4083 |
| **22/7.6** | IntraFamily | Repeatability | 0 | 0 |
|  | Animal | Heritability | 0 | 0 |
|  | Sire | Heritability | 0 | 0 |
|  | Dam | Heritability | 0 | 0 |
| **25/8.1** | IntraFamily | Repeatability | 0.5989 | 0.2681 |
|  | Animal | Heritability | 0.1926 | 0.4094 |
|  | Sire | Heritability | 0.3911 | 0.5657 |
|  | Dam | Heritability | 0.0272 | 0.3078 |
| **25/7.8** | IntraFamily | Repeatability | 0.8684 | 0.2079 |
|  | Animal | Heritability | 0 | 0 |
|  | Sire | Heritability | 0.5594 | 0.7079 |
|  | Dam | Heritability | 0 | 0 |
| **25/7.6** | IntraFamily | Repeatability | 0.7118 | 0.103 |
|  | Animal | Heritability | 0.3491 | 0.3280 |
|  | Sire | Heritability | 0.4223 | 0.5004 |
|  | Dam | Heritability | 0.2892 | 0.4205 |

Animal, sire and dam models were used to estimate heritability for fertilisation and gastrulation across each treatment. Multiple observations on the same genotype were included in the model as random effects and were used to compute repeatability. Temperature and pH were fixed effects and experimental block a random effect. The models were fitted using ASReml.
